# Monocarboxylate Transporters: Role and Regulation in Corneal Diabetes

**DOI:** 10.1155/2022/6718566

**Published:** 2022-10-26

**Authors:** Pawan Shrestha, Amy E. Whelchel, Sarah E. Nicholas, Wentao Liang, Jian-Xing Ma, Dimitrios Karamichos

**Affiliations:** ^1^North Texas Eye Research Institute, University of North Texas Health Science Center, 3430 Camp Bowie Blvd, Fort Worth, TX 76107, USA; ^2^Department of Pharmaceutical Sciences, University of North Texas Health Science Center, 3500 Camp Bowie Blvd, Fort Worth, TX 76107, USA; ^3^Department of Physiology, University of Oklahoma Health Sciences Center, 940 Stanton L Young Blvd, Oklahoma City, OK 73104, USA; ^4^Department of Biochemistry, Wake Forest University School of Medicine, 575 N Patterson Ave, Winston-Salem, NC 27101, USA; ^5^Department of Pharmacology and Neuroscience, University of North Texas Health Science Center, 3500 Camp Bowie Blvd, Fort Worth, TX 76107, USA

## Abstract

Diabetes mellitus (DM) is a group of metabolic diseases that is known to cause structural and functional ocular complications. In the human cornea, DM-related complications affect the epithelium, stroma, and nerves. Monocarboxylate transporters (MCTs) are a family of proton-linked plasma membrane transporters that carry monocarboxylates across plasma membranes. In the context of corneal health and disease, their role, presence, and function are largely undetermined and solely focused on the most common MCT isoforms, 1 through 4. In this study, we investigated the regulation of MCT1, 2, 4, 5, 8, and 10, in corneal DM, using established 3D self-assembled extracellular matrix (ECM) *in vitro* models. Primary stromal corneal fibroblasts were isolated from healthy (HCFs), type I (T1DMs), and type II (T2DMs) DM donors. Monoculture 3D constructs were created by stimulating stromal cells on transwells with stable vitamin C for two or four weeks. Coculture 3D constructs were created by adding SH-SY5Y neurons at two different densities, 12 k and 500 k, on top of the monocultures. Our data showed significant upregulation of MCT1 at 4 weeks for HCF, T1DM, and T2DM monocultures, as well as the 500 k nerve cocultures. MCT8 was significantly upregulated in HCF and T1DM monocultures and all of the 500 k nerve cocultures. Further, MCT10 was only expressed at 4 weeks for all cocultures and was limited to HCFs and T1DMs in monocultures. Immunofluorescence analysis showed cytoplasmic MCT expression for all cell types and significant downregulation of both MCT2 and MCT4 in HCFs, when compared to T1DMs and T2DMs. Herein, we reveal the existence and modulation of MCTs in the human diabetic cornea *in vitro*. Changes appeared dependent on neuronal density, suggesting that MCTs are very likely critical to the neuronal defects observed in diabetic keratopathy/neuropathy. Further studies are warranted in order to fully delineate the role of MCTs in corneal diabetes.

## 1. Introduction

Diabetes mellitus (DM) is a group of metabolic diseases that is known to cause structural and functional ocular complications. DM ocular complications are one of the leading causes of adult blindness in the world [1]. Both diabetic retinopathy and diabetic keratopathy/neuropathy are eminent risk factors for visual deterioration, resulting in more than 20,000 new cases of blindness each year [2]. In the human cornea, diabetic keratopathy/neuropathy is characterized by epithelial lesions, reduction in epithelial thickness, oxidative stress, inflammation, decreased corneal nerve densities, and declined corneal sensitivity. To date, the pathogenic mechanism of the diabetic cornea is not fully understood, representing a major knowledge gap and a clinical hurdle.

Monocarboxylate transporters (MCTs), also known as solute carrier (SLC16) transporter family members, are membrane proteins that are crucial in numerous mechanisms, including cell nutrients transportation, cellular metabolism, and pH regulation [3, 4]. MCTs play a key role in transporting the monocarboxylates such as lactate, pyruvate, and ketone bodies across the plasma membrane, which are essential in carbohydrate, fat, and amino acid metabolism [4–7]. In the context of ocular drug delivery, there are two different transporter systems: the efflux and influx transporters. Efflux transporters belong to the adenosine triphosphate (ATP) binding cassette superfamily, while influx transporters belong to the SLC superfamily [8]. To date, 14 MCT members (MCT1 through 14) have been identified as members of this SLC16 family [7, 9]. MCT isoforms 1 through 4, commonly identified as proton-dependent MCTs, are the most commonly studied, especially in the cancer field [4, 6, 9, 10]. MCTs 1-4 are often targeted in preclinical studies (e.g., highly glycolytic malignant tumors) using RNAi and small-molecule inhibitor alpha-cyano-4-hydroxycinnamic acid (CHC), targeting inhibition of pyruvate transport as a therapeutic strategy [9, 11]. The role of MCTs has also been investigated in obesity, fatigue, and type II DM, as well as diseases of the central and peripheral nervous systems [9, 12–16].

In the human cornea, to date, MCTs are critically understudied with only eleven studies published [4–7, 12, 17–22]. The majority of the existing literature focus on the expression of MCTs 1-4 [12, 18, 21] and MCT5 [19], as they relate to the corneal epithelium. Corneal endothelial cells have also been reported to express MCT1, MCT4, and MCT8 [17, 22]. It is well known that the diabetic cornea can suffer from lasting changes to the corneal epithelium, corneal nerves, stroma, endothelium, and conjunctiva, as well as corneal biomechanics and tear fluid [23, 24]. Importantly, a previous study on MCTs suggested that the clinical development of MCT1 inhibitors may lead to unexpected side effects and worsening of diabetic peripheral neuropathy (DPN) in type I DM patients [25]. Another study by Xu et al. has also shown that the expression and function of intestinal MCT6 are significantly downregulated in diabetic rats [26]. Thus, further studies are necessary in order to delineate the role of MCTs in the humans.

The aim of this study was to determine the existence and determine the modulation of MCTs in the healthy and diabetic corneal stroma, over time and in relation to nerve density. The coculture model used for this study could be a key step in better understanding the interaction between nerves and stromal cells in the context of the diabetic cornea. Given the impact of MCTs reported in various human tissues and organs, it is imperative to investigate their role in the human cornea and understand their therapeutic potential, if any, in corneal DM.

## 2. Materials and Methods

### 2.1. Ethical Approval, Consent, and Tissue Recovery

All studies adhered to the tenets of the Declaration of Helsinki. All experiments were performed after acquiring approval from the North Texas Regional Institutional Review Board (IRB) at the University of North Texas Health Sciences Center at Fort Worth (protocol #2020-030). Both healthy and diabetic corneal tissue samples were obtained from the National Disease Research Interchange (NDRI) and the Oklahoma Lions Eye Bank. The federal and institutional guidelines were followed while performing all the experiments described here. All human samples were deidentified prior to analysis.

### 2.2. Cell Isolation and Cultures

Primary human corneal fibroblasts were isolated from healthy, T1DM, and T2DM donor corneas, as previously reported [24]. Briefly, all human corneas were scraped to remove epithelium and endothelium, using sterile surgical scalpel blades [27]. The stromal tissue was then cut into small pieces, and explants were transferred into T25 flasks (4 or 5 pieces of ~2 × 2 mM per flask), followed by a 45-minute incubation at 37°C in 5% CO_2_ to allow adherence. Media was then carefully added containing Eagle's Minimum Essential Medium (EMEM: ATCC; Manassas, VA), 10% fetal bovine serum (FBS: Atlanta Biologicals, Flowery Branch, GA, USA), and 1% antibiotic-antimycotic (AA: Gibco, Grand Island, NY, USA). Within 1–2 weeks, the cells were passaged further into T75 flasks. Experiments were carried out using cells between P3 and P7, as previously established [28, 29]. The SH-SY5Y neuroblastoma cells (Sigma-Aldrich; St. Louis, MO) were cultured in similar conditions (EMEM, 10%FBS, 1%AA) and used in the coculture models, as well as immunofluorescence assays.

### 2.3. Experimental Groups

A total of six different conditions were analyzed. The monoculture groups consisted of “controls” HCFs (HCF-C), type 1DMs (T1DM-C), or type 2DMs (T2DM-C). The coculture groups consisted of innervated HCFs (HCF-N), type 1DMs (T1DM-N), or type 2DMs (T2DM-N). Cocultures were examined using two different nerve densities: 12 k and 500 k, to mirror nerve density changes seen in diabetic keratopathy/neuropathy. A single donor, per condition/disease, was used for the described studies. Both monocultures and cocultures were processed and analyzed at two different timepoints: 2 and 4 weeks, to mirror the corneal stroma changes seen *in vivo*.

### 2.4. 3D Constructs: Monocultures

HCFs, T1DMs, and T2DMs were seeded at a density of 1 × 10^6^/well on 0.4 *μ*M polycarbonate membranes (Corning, Corning, NY, USA), as previously described [27, 30–32]. Cultures were maintained in EMEM, 10% FBS, and 1% AA and further stimulated by 0.5 mM stable vitamin C (VitC; 0.5 mM 2-O-*α*-D-glucopyranosyl-L-ascorbic acid, Sigma-Aldrich, St. Louis, MO, USA) in order to promote secretion and assembly of extracellular matrix (ECM). The constructs were maintained in VitC media for 2 or 4 weeks with media changes occurring every other day [29].

### 2.5. 3D Constructs: Cocultures

Similar to the monocultures, all cocultures were maintained with VitC media for the duration of the experiments, as described previously [27, 33]. At the end of week 1 (for week 2 timepoints/cultures) and week 3 (for week 4 timepoints/cultures), 12 k or 500 k SH-SY5Ys were seeded directly on top of the assembled 3D stromal constructs per well. The lower well was supplemented with VitC medium, while the top well was treated with regular media (i.e., no VitC). After 24 h, the neuronal cells were treated with 10 *μ*M retinoic acid for 5 days in 1% FBS and 1% AA, followed by a 48-hour treatment of 2 nM brain-derived neurotrophic factor (BDNF) in serum-free medium to induce neuronal differentiation, as previously described [27, 33, 34]. Constructs were further processed at the end of weeks 2 and 4.

### 2.6. Immunofluorescence

HCFs, T1DMs, and T2DMs, as well as SH-SY5Y neurons, were plated on coverslips, in 12-well plates, at a cell density of 100 k per well (*n* = 4 each). Both nondifferentiated and differentiated SH-SY5Y neurons were tested. After 24 hours in culture, stromal cells and nondifferentiated SH-SY5Ys were fixed in 4% formaldehyde, as previously described [27, 35]. Briefly, fixed cells were permeabilized and blocked with 3% milk for 1 h, followed by overnight incubation with primary antibody: SLC16A1/MCT1(HPA003324; Sigma-Aldrich, St. Louis, MO, USA) 1 : 100, SLC16A7/MCT2 (ab224627; Abcam, Cambridge, UK) 1 : 200; SLC16A3/MCT4 (ab234728; Abcam, Cambridge, UK) 1 : 200; SLC16A4/MCT5 (SAB4301023; Sigma-Aldrich, St. Louis, MO, USA) 1 : 500; SLC16A2/MCT8 (HPA072719; Sigma-Aldrich, St. Louis, MO, USA) 1 : 100; and SLC16A10/MCT10 (HPA016860; Sigma-Aldrich, St. Louis, MO, USA) 1 : 100. Samples were then washed three times with PBS and incubated for 1 h with Alexa Fluor secondary antibodies at 1 : 2000 (Thermo Fisher Scientific, Waltham, MA, USA). Samples were washed again, three times with PBS, before being stained with DAPI (nuclei stain) and mounted on glass slides. A similar procedure was followed for the differentiated SH-SY5Y neurons where they were treated with 10 *μ*M retinoic acid for 5 days in 1% FBS and 1% AA, followed by a 48-hour treatment of 2 nM brain-derived neurotrophic factor (BDNF). All images were captured by Keyence BZ-X710 All-in-one Fluorescence Microscope (Itasca, IL, USA) and analyzed using BZ-X Analyzer and ImageJ [36].

### 2.7. RNA Isolation

Total RNA, from all 3D constructs, was extracted using the Ambion RNA mini extraction kit, as previously described (Ambion TRIzol® Plus RNA Purification Kit: Life Technologies, Carlsbad, CA) [37]. Briefly, the culture media was removed from all cultures, followed by phosphate-buffered saline (PBS) wash. The cells were then gently scraped from the membrane with forceps and transferred to a tube with 1 mL of TRI Reagent® (Life Technologies Corporation, Carlsbad, CA). Chloroform was added to create a phase separation, and total RNA contained in the aqueous phase was purified using the RNeasy® mini kit column (QIAGEN, Hilden, Germany) according to the manufacturer's protocol. Ultraviolet spectrometer (Epoch 2, BioTek Instruments Inc., Agilent, Santa Clara, CA, USA) was used to evaluate the purity and quantity of the total RNA obtained [38].

### 2.8. Real-Time PCR

Real-time PCR was performed following cDNA synthesis, using a SuperScript III First-Strand Synthesis SuperMix (Invitrogen, Carlsbad, CA) according to the manufacturer's protocol. All reactions were performed using 10 ng of cDNA in a 10 *μ*L reaction containing the target probe and TaqMan Fast Advanced Master Mix (Applied Biosystems, Life Technologies, Foster City, CA). Real-time PCR was performed on a QuantStudio™ 3 Real-Time PCR System (Applied Biosystems; Thermo Fisher Scientific, Waltham, MA, USA). GAPDH (Hs99999905_m1) and 18S (Hs99999901_s1) were used as housekeeping. The following MCT probes were tested: SLC16A1/MCT1 (Hs01560299_m1), SLC16A7/MCT2 (Hs00940851_m1), SLC16A3/MCT4 (Hs00358829_m1), SLC16A5/MCT5 (Hs04187570_m1), SLC16A2/MCT8 (Hs00185140_m1), and SLC16A10/MCT10 (Hs01039921_m1) (Thermo Fisher Scientific; Rockford, IL, USA). GraphPad Prism 9 and MS-Excel were used for data analysis. All samples and targets were repeated at least four times [39].

### 2.9. Statistical Analysis

GraphPad Prism 9 was used for all statistical analyses. One-way ANOVA was used to conduct the statistical data analysis and *p* < 0.05 was considered statistically significant. Data obtained in triplicates with each set were repeated at least 4 times per cell type/condition.

## 3. Results

### 3.1. Immunofluorescence Showing the Presence of Different MCT Isoforms

**Figure 1:**
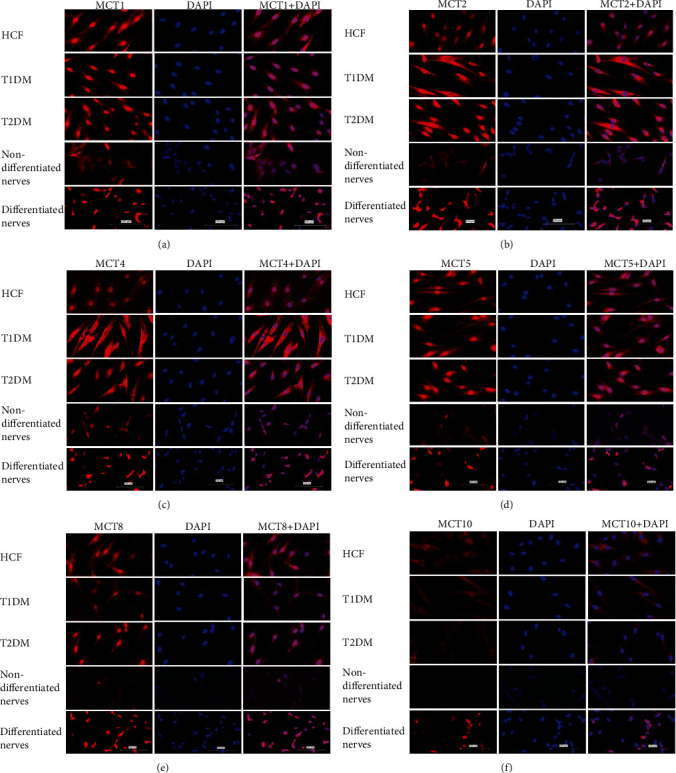
Immunofluorescence staining for HCFs, T1DMs, and T2DMs along with differentiated and nondifferentiated nerves with MCT markers (red) in 2D cell culture. Nuclei were stained with Dapi (blue) and their overlap was seen (a–f). Scale bars: 100 *μ*M.

Immunofluorescence of all 2D cultures investigated the expression of all MCTs. All four cell types (HCFs, T1DMs, T2DMs, and differentiated and nondifferentiated SH-SY5Ys) were investigated. Immunofluorescence intensity was captured for all MCT markers, and DAPI was used as nuclei stain (negative controls, see Supplement Figure 1).  Figure 1 shows the presence of all MCTs on all cell types tested: HCFs, T1DMs, T2DMs, nondifferentiated nerves, and differentiated nerves.

### 3.2. MCT Gene Expression

#### 3.2.1. Cocultures with 12 k Nerve Density

**Figure 2:**
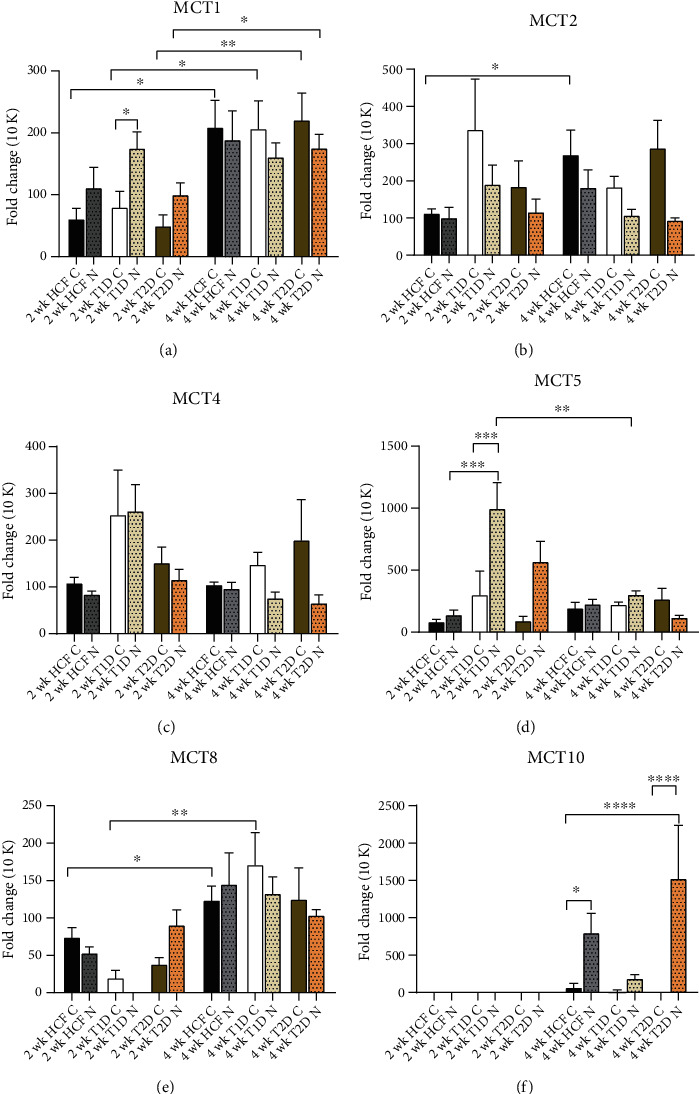
MCT marker expression for HCF, T1DM, and T2DM controls and their cocultures with 12 k cell density over the time interval of 2 weeks and 4 weeks. Gene expression quantification normalized to their controls for each cell type. Error bars are used to represent the standard error of the mean. One-way ANOVA was performed (^∗^*p* < 0.05, ^∗∗^*p* < 0.01, ^∗∗∗^*p* < 0.001, and ^∗∗∗∗^*p* < 0.0001).

MCT gene expression from monocultures and cocultures were investigated using real-time PCR.  Figure 2(a) shows significant upregulation of MCT1 for HCF-C, T1DM-C, and T2DM-C along with 12 k T2DM-N at 4 weeks, as compared to that of 2 weeks. MCT1 was also significantly upregulated with 12 k T1DM-N when compared to its monoculture equivalent at 2 weeks (Figure 2(a)). MCT2 was significantly upregulated only with the HCF-C at 4 weeks (Figure 2(b)). No significance was observed with MCT4 (Figure 2(c)). MCT5 was significantly downregulated in 12 k T1DM-N at 4 weeks compared to 2 weeks (Figure 2(d)). MCT8 was significantly upregulated in HCF-C and T1DM-C at 4 weeks compared to 2 weeks (Figure 2(e)). Interestingly, no expression was detected for MCT10 at week 2, for neither of the cell types with monocultures or cocultures. MCT10 expression was, however, apparent and significantly upregulated in week 4 HCF and T2DM cocultures (Figure 2(f)).

#### 3.2.2. Cocultures with 500 k Nerve Density

**Figure 3:**
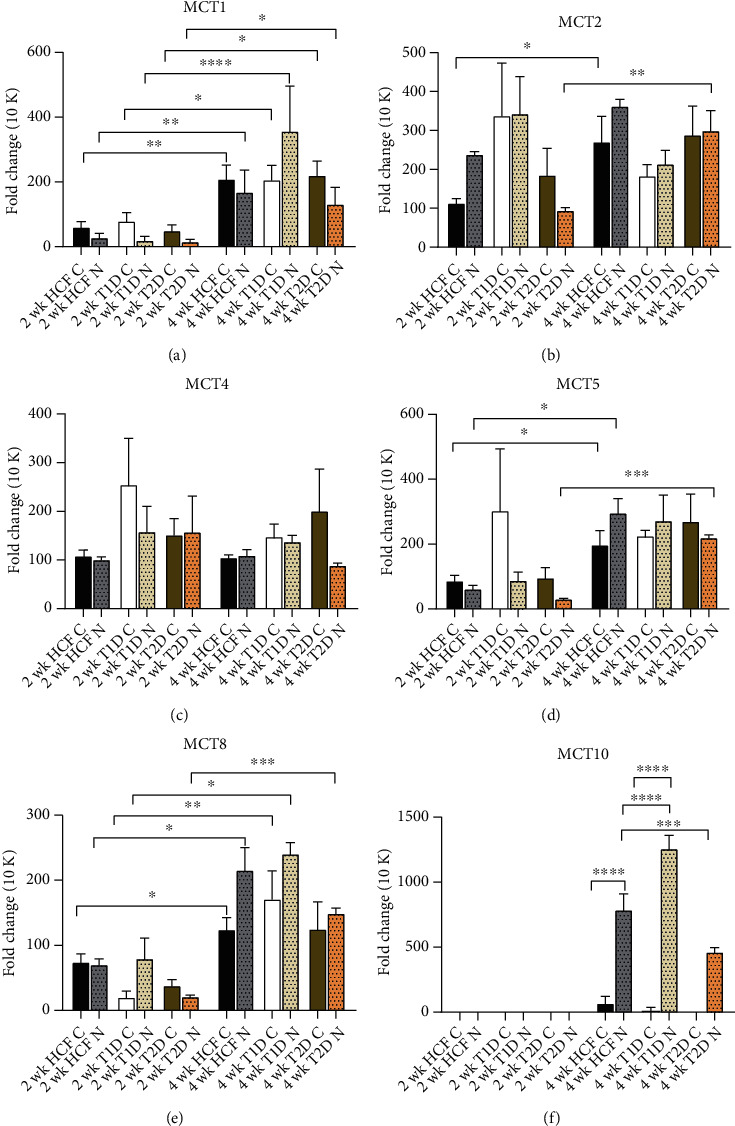
MCT marker expression for HCF, T1D, and T2D controls and their cocultures with 500 k cell density over the time interval of 2 weeks and 4 weeks. Gene expression quantification normalized to their controls for each cell type. Error bars are used to represent the standard error of the mean. One-way ANOVA was performed (^∗^*p* < 0.05, ^∗∗^*p* < 0.01, ^∗∗∗^*p* < 0.001, and ^∗∗∗∗^*p* < 0.0001).

The expression of different MCTs was also investigated in cocultures with higher nerve density, 500 k. MCT1 was significantly upregulated for HCFs, T1DMs, and T2DMs monocultures along with cocultures at 4 weeks (Figure 3(a)). MCT2 was significantly upregulated only for HCF-C and 500 k T2DM-N at 4 weeks (Figure 3(b)). No significance was observed for MCT4 (Figure 3(c)), while the HCF-C, 500 k HCF-N, and 500 k T2DM-N were significantly upregulated at 4 weeks for MCT5 (Figure 3(d)). HCF-C and T1DM-C along with cocultures of all three cell types at 4 weeks showed significantly upregulated expression of MCT8 (Figure 3(e)). 500 k HCF-N and 500 k T1DM-N at 4 weeks showed significantly upregulated MCT10, while no expression was observed at 2 weeks (Figure 3(f)).

## 4. Discussion

Recently, DM has reached epidemic proportions and is currently the leading cause of new blindness in adults [2, 24, 34, 40–43]. The human cornea is significantly affected by DM, a disease known as diabetic keratopathy/neuropathy, with complications observed in 45-70% of the diabetic population [40, 42, 44, 45]. Unfortunately, despite extensive research, the pathobiology and molecular mechanisms are not fully understood.

As previously reviewed by Shah et al. and Ljubimov et al. [24, 41], clinically observed corneal diabetic changes include epithelial defects with recurrent erosions, edema, superficial punctate keratitis, delayed wound repair/healing, endothelial changes, and neuropathy with notable reduction of corneal sensitivity [42, 46–58]. Further, diabetics also suffer from low tear secretion and dry eye syndrome [59–63], as well as dyslipidemia with increased content of sphingosines and ceramides [45, 64]. There remains a need for better understanding of the diabetic cornea pathobiology.

MCTs are known for their transport of metabolic products like lactate and pyruvate across the cell membrane [3, 21]. Their importance has been investigated in a wide range of tissues; however, most of the studies focus on MCT1, 2, 3, and/or 4, as they are the proton-dependent transporters of monocarboxylic acid [65]. The MCTs 1-4 have also been investigated in human corneal studies. MCT1 and MCT4 were found in the corneal epithelium, possibly assisting in ocular absorption of monocarboxylic acid drugs. Specifically, MCT1 was found to be >100-fold higher in primary human corneal epithelial cells than that in the freshly isolated tissue, whereas MCT4 was only found to be 5-10-fold higher [21, 66]. On the other hand, MCT3 was weakly detected in the corneal epithelium and primary cells [21]. The presence of MCT4 in the human corneal epithelium (21) is in contrast to our observations in the corneal stroma where we observed no expression of MCT4. Furthermore, MCTs 1-5 are found in rabbit corneal epithelial cells (RCECs), with the expression of MCT1, MCT4, and MCT5 found on the surface layer [19]. MCT2 was clearly localized in RCECs, and its expression was significantly higher, in both the rat corneal epithelium and endothelium, compared to other MCTs [19, 66]. It is feasible that modulation of MCTs varies among species; however, further studies are needed in order to determine the translatability of *in vivo* findings.

MCT1 is known to be the most ubiquitous followed by MCT4 with both present in most tissues [3, 67]. MCT3 is found mostly in the retinal pigment epithelium whereas MCT2 has more restrictive distribution and is expressed in the testis and the intracellular membrane of neuronal mitochondria [68]. MCTs 1-4 are mostly known for their transport of monocarboxylates, which plays a major role in the metabolic communication between cells. MCT5, on the other hand, is found in the placenta, and a recent study has shown it to be significantly upregulated in colorectal adenocarcinoma, which could suggest its probable importance in gastrointestinal caner [3, 69, 70]. Outside of MCTs 1-4, MCT8 is the next most well-studied MCT, influencing thyroid hormone transport. A mutation in this gene is known to cause a rare X-linked neurologic disorder, called Allan-Herndon-Dudley syndrome (AHDS) [3], with patients experiencing abnormal eye movement (nystagmus). MCT8 helps in the transport of thyroid hormones along with MCT10, which is mostly found in chondrocytes [3, 71]. Studies have reported that both, MCT 8 and MCT 10, are present in the human hypothalamus by weeks 17 and 25 of gestation [3, 72]. Disruption in the function of MCT10 was shown to cause significant problems to the endochondral ossification process [73].

In our study, we investigated the most common MCT1, MCT2, and MCT4, along with MCT5, 8, and 10, using corneal stromal cells (healthy and diabetic donors) and neuroblastoma cells (SH-SY5Y). The goal was to characterize MCT expression and determine their impact on corneal stroma and corneal DM. Further, utilizing a coculture system, we tested the MCT modulation in relation to the stroma-nerve interactions.

Our findings revealed significant upregulation of MCT1, MCT8, and MCT10 expressions in our nerve cocultures for all cell types and timepoints. MCT10 was surprisingly only expressed at 4 weeks for both mono- and cocultures, suggesting a possible late onset or a homeostatic dependence. MCT10 alone, probably, warrants further investigation in order to accurately delineate its role in the cornea. The expression of MCT1 that we observed in corneal stromal cells only validates its ubiquitous presence in the body. MCT1 has been found to be expressed in almost all tissues. It is because of its ubiquitous nature that it is found to be involved in variety of diseases. The modulation of the different MCTs seen here with thinner and thicker stromal ECMs (2 vs. 4 weeks) and nerve densities (12 vs 500 k) further underscores the potential role of each MCT isoform in corneal DM.

The relatively narrow and unjustified focus on MCT isoforms 1 through 4, despite the 14 identified isoforms, highlights the need for more comprehensive and more inclusive studies in order to determine what the exact role for MCTs is.

## 5. Conclusions

Our study shows the presence of MCTs in the context of the diabetic cornea. The changes observed, due to neuronal density, suggest that MCTs are likely critical to neuronal degeneration and pathology seen in diabetic keratopathy/neuropathy. Further studies are warranted, in order to fully delineate the role of MCTs in the human cornea. Overall, our study not only provides a new platform for MCT-corneal research but also provides new insight into the limited studies investigating T1DM versus T2DM corneal manifestations.

## Data Availability

Data is available upon request from the corresponding author.

## Supplementary Materials

Supplementary Materials The following supporting information was supplied as separate files. Supplementary Figure 1: immunofluorescence staining for HCFs, T1DMs, and T2DMs along with nerves pre- and postdifferentiation “negative controls” in 2D cell culture. Scale bars: 100 *μ*M.
