# Supplementary material for: Monocarboxylate Transporters: Role and Regulation in Corneal Diabetes
Source: Anal Cell Pathol (Amst). 2022 Oct 26;2022:6718566. doi: 10.1155/2022/6718566 (PMC9629935; doi:10.1155/2022/6718566)
Supplement: Supplementary Materials — The following supporting information was supplied as separate files. Supplementary Figure 1: immunofluorescence staining for HCFs, T1DMs, and T2DMs along with nerves pre- and postdifferentiation “negative controls” in 2D cell culture. Scale bars: 100 μM. [file 6718566.f1.docx]

**Supplemental Materials:**


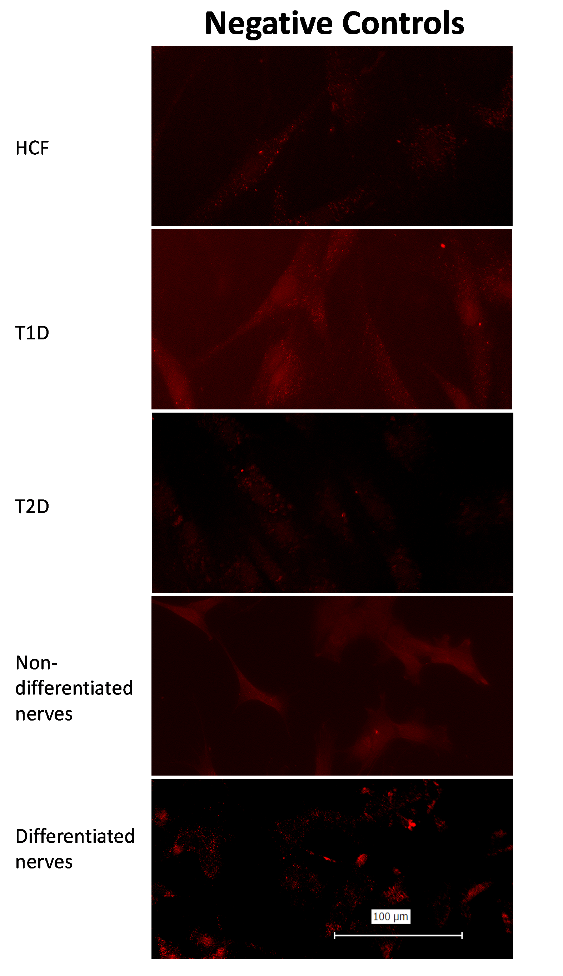


**Supplemental Figure 1.** Immunofluorescence staining for HCFs, T1DMs, T2DMs along with differentiated and non-differentiated nerves, “negative controls” in 2D cell culture. Scale bars: 100 µm.
